# Associations of apolipoprotein B/apolipoprotein A-I ratio with pre-diabetes and diabetes risks: a cross-sectional study in Chinese adults

**DOI:** 10.1136/bmjopen-2016-014038

**Published:** 2017-01-20

**Authors:** Shuang Zheng, Tingting Han, Hua Xu, Huan Zhou, Xingxing Ren, Peihong Wu, Jun Zheng, Lihua Wang, Ming Zhang, Yihong Jiang, Yawen Chen, Huiying Qiu, Wei Liu, Yaomin Hu

**Affiliations:** Department of Endocrinology, Renji Hospital, School of Medicine, Shanghai Jiaotong University, Shanghai, China

**Keywords:** apolipoprotein B, apolipoprotein A-I, type 2 diabetes, prediabetes, dyslipidemia

## Abstract

**Background:**

Apolipoprotein B/apolipoprotein A-I (ApoB/ApoA-I) ratio is a useful predictor of cardiovascular risk. However, the association between the ApoB/ApoA-I ratio and the risk of type 2 diabetes mellitus (T2DM) is still obscure.

**Aims:**

To investigate the associations between the ApoB/ApoA-I ratio and the risk of T2DM and pre-diabetes in a Chinese population, and to assess the role of gender in these associations.

**Methods:**

A stratified random sampling design was used in this cross-sectional study which included 264 men and 465 women with normal glucose tolerance (NGT), pre-diabetes or T2DM. Serum ApoB, ApoA-I and other lipid and glycaemic traits were measured. Pearson's partial correlation and multivariable logistic analysis were used to evaluate the associations between ApoB/ApoA-I ratio and the risk of T2DM and pre-diabetes.

**Results:**

The ApoB/ApoA-I ratios were significantly increased across the spectrum of NGT, pre-diabetes and T2DM. Women showed higher levels of ApoB/ApoA-I ratio and ApoB than men in the pre-diabetic and T2DM groups, but not in the NGT group. The ApoB/ApoA-I ratio was closely related with triglyceride, total cholesterol, high-density lipoprotein cholesterol, low-density lipoprotein cholesterol and other glycaemic traits. Moreover, in women, the risk of diabetes and pre-diabetes in the top and middle tertiles of the ApoB/ApoA-I ratio were 3.65-fold (95% CI 1.69 to 6.10) and 2.19-fold (95% CI 1.38 to 2.84) higher than in the bottom tertile, respectively, after adjusting for potential confounding factors. However, the associations disappeared in men after adjusting for other factors.

**Conclusions:**

The ApoB/ApoA-I ratio showed positive associations with the risk of diabetes and pre-diabetes in Chinese women.

**Strengths and limitations of this study**

- This is an in-depth investigation of the associations between the apolipoprotein B/apolipoprotein A-I (ApoB/ApoA-I) ratio and diabetes or pre-diabetes in a Chinese population.
- Stratified random sampling ensured an equal chance for participant enrolment and the validity of results.
- Data collection and analysis were supported by a trained survey team, which included researchers with differing areas of expertise and backgrounds.
- The cross-sectional design does not confirm the causality between ApoB/ApoA-I ratio and diabetes risk.
- Potential bias might exist in a single-centre design.

## Introduction

Atherogenic dyslipidaemia is a characteristic lipoprotein abnormality that includes high levels of triglyceride (TG)-rich lipoproteins (mainly very low-density lipoprotein (VLDL)), low levels of high-density lipoproteins (HDL) and an elevated proportion of small dense low-density lipoprotein (sd-LDL) particles.^1^ To date, evaluation of the clinical relevance of atherogenic dyslipidaemia has focused on its role as an effective predictor of cardiovascular disease (CVD) compared to other lipid parameters. Apolipoproteins are important structural and functional proteins in lipoprotein particles. Given that circulating levels of apolipoproteins indicate the number of lipoprotein particles, the level of apolipoprotein B (Apo-B) reflects the total number of potentially atherogenic particles, including VLDL, intermediate density lipoproteins, large buoyant LDL, and sd-LDL, and the level of apolipoprotein A-I (ApoA-I) represents the number of HDL particles.^2^ Thus, the ratio of Apo-B to ApoA-I (ApoB/ApoA-I) would theoretically be an ideal indicator of atherogenic lipid disturbances and cardiovascular risk.^3^ ^4^

It is well established that atherogenic dyslipidaemia is associated with type 2 diabetes mellitus (T2DM) and a high risk of CVD in T2DM patients.^5^ ^6^ Moreover, the mortality rate of CVD in diabetic patients varies with gender.^7^ Previous studies have demonstrated that the ApoB/ApoA-I ratio may be a strong marker of metabolic syndrome and insulin resistance in certain population,^8–10^ but the associations of it with T2DM and pre-diabetes as well as its gender effects in the Chinese population are still poorly clarified. Additionally, the correlations between ApoB/ApoA-I ratio and other lipid or glycaemic traits, such as TG, total cholesterol (TC), glucose, insulin sensitivity and secretion, need further investigation.

Therefore, in this observational study, we aimed to evaluate the associations between the level of ApoB/ApoA-I ratio and the risk of T2DM and pre-diabetes in both Chinese men and women and also investigate the correlations between the ratio and other lipid and glycaemic traits.

## Subjects and methods

### Subjects

Stratified random sampling was performed to select participants from the database of Renji Hospital from January 2010 to December 2014. A total of 1538 subjects aged 18–80 years were included. All of them had visited the Department of Endocrinology, Renji Hospital, School of Medicine, Shanghai Jiaotong University for a health check-up.

The exclusion criteria were regular diabetic and/or lipid-lowing medication use, a history of CVD, cerebrovascular disease, chronic renal or hepatic failure, cancer, pregnancy, hyperthyroidism and hypothyroidism. Subjects with incomplete data were also excluded.

Finally, 729 subjects (264 men and 465 women) were involved in this study. The study was carried out in accordance with the declaration of Helsinki and the study protocol was approved by the Ethical Committee of Renji Hospital, School of Medicine, Shanghai Jiaotong University. Written informed consent was obtained from all subjects included in the study.

### Anthropometry measurements

Height, body weight, waist circumference (WC), hip circumference and blood pressure (BP) were measured by trained survey personnel. Height was measured once with a portable height scale to the nearest 0.1 cm. Weight was measured using a platform digital scale to the nearest 0.1 kg. Both height and weight measurements were taken in light clothing without shoes. WC was recorded as the circumference midpoint between the iliac crest and the lowest rib. Hip circumference was recorded as the largest gluteal circumference. Circumference measurements were taken twice by a single observer and the mean value was reported. BP was measured twice in each subject on the right arm after a 5 min rest in a sitting position, and the mean of two measurements was used.

### Laboratory analysis

All subjects underwent the standard 75 g glucose oral glucose tolerance test (OGTT) after an 8-hour overnight fast. Serum ApoB (predominantly ApoB-100) and ApoA-I concentrations were determined by immunoturbidimetric methods using an automatic immunoassay analyser (Roche E-170, Roche, Basel, Switzerland). Glucose and other lipid levels were measured using fully automatic biochemistry analysers (Hitachi 7600–110 and Hitachi 7020, respectively, Hitachi Co. Tokyo, Japan); insulin concentration was determined by an immunoradiometric assay kit (Dainabot, Tokyo, Japan); and haemoglobin A1c (HbA1c) level was measured by high-performance liquid chromatography.

### Definition of T2DM and pre-diabetes

According to the 2006 WHO criteria,^11^ diabetes was defined as fasting plasma glucose (FPG) ≥7.0 mmol/L and/or 2-hour post-load plasma glucose (2hPG) ≥11.1 mmol/L; pre-diabetes was defined as FPG between 6.1 and 7.0 mmol/L and/or 2hPG between 7.8 and 11.1 mmol/L. For diabetes, the diagnosis was predominantly based on the results of the 75 g OGTT. In addition, a level of glycated haemoglobin (HbA1c) >6.5% was also considered as a supplementary criterion. If a subject met both criteria, then the diagnosis of diabetes was certain. However, if a subject met the criterion of OGTT but the level of HbA1c was <6.5%, then the OGTT would be repeated within 1 month. Two abnormal measurements of OGTT confirmed the diagnosis of diabetes. If a subject did not meet the criterion of OGTT at the first time, then he or she would be excluded. For pre-diabetes, the diagnosis would be ensured with repeated OGTT in 1 month. Anyone who did not meet the criterion of OGTT at the second time would be excluded. Subjects who declined to take the second examination were also excluded.

### Calculation

Body mass index (BMI) was defined as the body weight (kg) divided by the body height squared (m^2^). Waist to hip ratio (WHR) was calculated as WC (cm) divided by hip circumference (cm). Indices of insulin resistance and insulin secretion were calculated from the OGTT data: homeostasis model assessment for insulin resistance (HOMA-IR)=fasting insulin (μU/mL)×fasting glucose (mmol/L)/22.5; homeostasis model assessment for β cell function index (HOMA-β)=20×fasting insulin in μU/mL / (fasting glucose in mmol/L −3.5).

### Statistical analysis

All statistical analyses were performed using SPSS V.17.0 (SPSS, Chicago, Illinois, USA). The available-case analysis (pairwise deletion) was applied to handle missing data. Continuous data were expressed as median (IQR 25–75%) due to the skewed distribution and compared by Kruskal-Wallis H test or Mann-Whitney U test. Adjusted means were calculated and compared with general linear models. Categorical variables were shown as percentages and compared with χ^2^ test. A Pearson's partial correlation analysis was carried out to identify the correlativity between ApoB/ApoA-I ratio and other variables after adjusting for several covariates. Data with skewed distribution were log-transformed before analysis. A multivariable logistic regression model was conducted to test the associations between ApoB/ApoA-I ratio and the risk of pre-diabetes and diabetes after controlling for potential confounding factors. The relative ratios (RRs) and 95% CIs of tertiles 2 to 3 were calculated and compared by using tertile 1 as reference. RR was calculated based on the formula: RR=OR/(1−P0+P0×OR), where OR was odds ratio and P0 was the disease incidence in the non-exposed group. Statistical significance was considered at p<0.05.

## Results

### Clinical and laboratory characteristics

After excluding ineligible subjects on the basis of exclusion criteria and those unable to finish the study, 729 subjects were finally included in this study. Among these eligible participants, 36.2% were men and 63.8% were women, with a mean age of 51.2 years; 30.5% of subjects (28.4% men and 31.6% women) were diagnosed as having T2DM, 32.9% (34.1% men and 32.3% women) as having pre-diabetes, and 36.6% (37.5% men and 36.1% women) as having normal glucose tolerance (NGT).

The anthropometric and metabolic characteristics of the participants are presented in table 1. Subjects with T2DM had much higher ApoB/ApoA-I ratios than those with pre-diabetes and NGT, accompanied by worse glucose and lipid profiles. Moreover, T2DM subjects showed higher levels of BP, BMI and WHR. Subjects with pre-diabetes had higher ApoB/ApoA-I ratios than those with NGT. The levels of BP, BMI and WHR in the pre-diabetic group were also higher than those with NGT.

**Table 1:** Characteristics of participants according to glucose status and gender

	NGT			Pre-diabetes			Diabetes					
	Total	Men	Women	Total	Men	Women	Total	Men	Women	*P*1	*P*2	*P*3
Number	267	99	168	240	90	150	222	75	147	NS	NS	NS
Age (years)	51.0 (41.0– 57.0)	51.0 (41.5– 57.5)	51.0 (40.0– 57.0)	56.0 (45.0–61.0)	56.0 (44.0– 59.0)	56.0 (47.0– 62.0)	56.0 (46.0– 60.0)	58.0 (46.0–61.5)	56.0 (46.0– 59.5)	<0.001	<0.001	NS
SBP (mm Hg)	114.0 (113.0– 116.0)	114.0 (102.0– 127.0)	110.0 (102.0– 120.5)	121.0 (119.0– 123.0)	116.0 (110.0– 131.0)	121.0* (114.0– 132.0)	121.0 (119.0– 123.0)	119.0 (113.0– 129.5)	120.0 (110.0– 130.5)	<0.001	<0.001	NS
DBP (mm Hg)	74.0 (73.0– 75.0)	72.0 (68.0– 79.0)	74.0 (69.0– 80.0)	77.0 (76.0– 78.0)	78.0 (72.0– 83.0)	78.0 (74.0– 82.0)	80.0 (78.0– 81.0)	79.0 (73.5– 82.0)	79.0 (75.5– 85.5)	<0.001	<0.001	0.005
BMI	23.03 (22.60– 23.46)	23.11 (21.32– 25.15)	23.06 (19.78– 24.92)	23.92 (23.47– 24.37)	24.12 (21.21– 27.46)	23.64 (21.79– 26.33)	25.09 (24.61– 25.55)	27.67 (24.23– 29.45)	23.37** (21.94– 26.38)	0.005	<0.001	<0.001
WHR	0.86 (0.85– 0.87)	0.88 (0.82– 0.91)	0.85* (0.81– 0.90)	0.89 (0.88– 0.89)	0.89 (0.84–0.93)	0.89 (0.86– 0.92)	0.90 (0.90– 0.91)	0.91 (0.89– 0.94)	0.90* (0.87– 0.93)	<0.001	<0.001	0.001
FPG (mmol/L)	4.98 (4.87– 5.09)	4.98 (4.65– 5.25)	5.01 (4.75– 5.35)	5.57 (5.46– 5.69)	5.44 (4.92– 5.97)	5.60 (5.04– 6.11)	7.31 (7.19– 7.43)	7.33 (7.16– 8.06)	7.15** (5.73– 7.87)	<0.001	<0.001	<0.001
2hPG (mmol/L)	6.01 (5.77– 6.26)	5.88 (5.30– 6.55)	6.05 (5.19– 6.83)	9.29 (9.03– 9.54)	9.19 (8.51– 9.85)	9.36 (8.58– 10.19)	13.95 (13.68– 14.21)	12.99 (12.23– 14.76)	13.07 (11.92– 14.73)	<0.001	<0.001	<0.001
FINS (μIU/L)	13.73 (12.85– 14.61)	12.27 (9.51– 16.90)	11.98 (8.76– 20.11)	14.70 (13.78– 15.61)	11.75 (10.59– 17.71)	12.06 (10.74– 18.71)	15.38 (14.42– 16.33)	14.77 (10.80– 20.03)	14.19 (9.82– 18.29)	NS	0.014	NS
2hINS (μIU/L)	64.18 (57.49– 70.87)	42.98 (24.02– 87.40)	55.13 (35.57– 71.38)	80.05 (73.08– 87.02)	75.27 (41.60– 119.56)	71.97 (48.35– 103.81)	71.23 (63.97– 78.49)	65.53 (24.57– 90.22)	63.75* (34.21– 110.37)	0.001	NS	NS
HbA1c(%)	5.5 (5.4– 5.6)	5.5 (5.2– 5.7)	5.6 (5.3– 5.8)	5.8 (5.7– 5.9)	5.8 (5.5– 6.0)	5.9* (5.6– 6.1)	6.9 (6.8– 7.0)	6.9 (6.6– 7.3)	6.7 (6.0– 7.4)	<0.001	<0.001	<0.001
HOMA-IR	3.07 (2.81– 3.33)	2.63 (2.01– 3.86)	2.67 (1.95– 4.60)	3.72 (3.46– 3.99)	2.93 (2.27– 4.18)	3.28 (2.58– 4.58)	5.04 (4.76– 5.32)	4.82 (3.46– 7.29)	4.18 (3.15– 5.85)	0.001	<0.001	<0.001
HOMA-β	218.63 (203.73– 233.53)	185.08 (136.51– 246.24)	161.87 (124.18– 257.26)	154.67 (139.14– 170.19)	123.17 (94.47– 185.50)	122.29 (87.07– 186.75)	93.41 (77.24– 109.58)	77.45 (58.17– 88.08)	74.54 (55.07– 112.40)	<0.001	<0.001	<0.001
TG (mmol/L)	1.50 (1.36– 1.65)	1.23 (0.77– 1.70)	1.15 (0.77– 1.77)	1.77 (1.62– 1.92)	1.50 (1.06– 1.99)	1.68 (1.09– 2.24)	2.72 (2.56– 2.88)	2.55 (1.73– 4.14)	2.44 (1.69– 3.50)	0.012	<0.001	<0.001
TC (mmol/L)	5.02 (4.92– 5.12)	4.94 (4.67– 5.20)	5.02* (4.67– 5.40)	5.13 (5.03– 5.23)	5.15 (4.31– 5.78)	5.27 (4.66– 5.55)	5.64 (5.53– 5.75)	5.47 (5.13– 6.18)	5.90 (4.93– 6.29)	NS	<0.001	<0.001
HDL-C (mmol/L)	1.48 (1.44– 1.51)	1.43 (1.19– 1.69)	1.45 (1.24– 1.68)	1.34 (1.31– 1.38)	1.30 (1.14– 1.48)	1.29 (1.15– 1.46)	1.23 (1.19– 1.27)	1.12 (1.09– 1.28)	1.28* (1.07– 1.38)	<0.001	<0.001	<0.001
LDL-C (mmol/L)	3.03 (2.95– 3.12)	2.84 (2.58– 3.29)	3.12 (2.58– 3.45)	3.05 (2.96– 3.14)	2.83 (2.60– 3.78)	3.08 (2.76– 3.54)	3.37 (3.28– 3.47)	3.50 (2.88– 3.69)	3.36 (3.02– 4.18)	NS	<0.001	<0.001
ApoA-I (g/L)	1.48 (1.45– 1.51)	1.43 (1.31– 1.68)	1.45 (1.28– 1.63)	1.39 (1.36– 1.43)	1.40 (1.26– 1.56)	1.34 (1.25– 1.52)	1.33 (1.29– 1.36)	1.36 (1.22– 1.38)	1.31 (1.17– 1.42)	<0.001	<0.001	0.005
ApoB (g/L)	0.84 (0.81– 0.86)	0.80 (0.68– 0.97)	0.84 (0.68– 0.97)	0.91 (0.88– 0.93)	0.90 (0.70– 0.98)	0.97** (0.84– 1.03)	0.97 (0.94– 1.00)	0.97 (0.80– 1.03)	1.05** (0.87– 1.17)	<0.001	<0.001	0.001
ApoB/ApoA-I ratio	0.60 (0.57– 0.62)	0.55 (0.42– 0.72)	0.56 (0.44– 0.72)	0.67 (0.64– 0.70)	0.66 (0.48– 0.77)	0.74** (0.56– 0.81)	0.76 (0.73– 0.78)	0.71 (0.60– 0.82)	0.78** (0.68– 0.94)	<0.001	<0.001	<0.001

Data are expressed as median (IQR 25–75%).

*P*1: NGT versus pre-diabetic group, *P*2: NGT versus diabetic group, *P*3: pre-diabetic versus diabetic group.

*p<0.05, **p<0.01 means the gender difference in each group.

Comparisons among NGT, pre-diabetic and diabetic groups were performed after adjusting for age.

2hINS, 2 hour postload serum insulin; 2hPG, 2 hour postload plasma glucose; ApoA-I, apolipoprotein A-I; ApoB, apolipoprotein B; BMI, body mass index; DBP, diastolic blood pressure; FINS, fasting serum insulin; FPG, fasting plasma glucose; HbA1c, glycated haemoglobin A1c; HDL-C, high-density lipoprotein cholesterol; HOMA-IR, homeostatic model assessment of insulin resistance; HOMA-β, homeostatic model assessment of β-cell function; LDL-C, low-density lipoprotein cholesterol; NGT, normal glucose tolerance; NS, not significant; SBP, systolic blood pressure; TC, total cholesterol; TG, triglyceride; WHR, waist to hip ratio.

When men and women were analysed separately, the ApoB/ApoA-I ratio in women was higher than in men both in the T2DM and pre-diabetic groups. However, the gender difference in the NGT group was not significant. Additionally, the levels of ApoB in women were higher than in men in both the T2DM and pre-diabetic groups. However, other lipid indices, including TG, TC and LDL cholesterol (LDL-C), showed little difference between men and women.

### Correlations between ApoB/ApoA-I ratio and other variables

After adjusting for age, systolic BP (SBP) and diastolic BP (DBP), Pearson's partial correlation analysis showed that the ApoB/ApoA-I ratio was positively correlated with TG, TC and LDL-C and negatively correlated with HDL cholesterol (HDL-C), in all three groups (table 2). Gender difference was insignificant. In addition, the ApoB/ApoA-I ratio was strongly associated with FPG, HbA1c and HOMA-IR both in men and women. However, the correlations between ApoB/ApoA-I ratio and HOMA-β, were not significant.

**Table 2:** Partial correlation coefficients of apolipoprotein B/apolipoprotein A-I (ApoB/ApoA-I) ratio with lipid profiles and glycometabolism-related traits

	NGT		Pre-diabetes		Diabetes	
	Men	Women	Men	Women	Men	Women
TG (mmol/L)	0.500***	0.474***	0.560***	0.570***	0.503***	0.654***
TC (mmol/L)	0.410***	0.350***	0.331**	0.271**	0.292*	0.648***
HDL (mmol/L)	−0.835***	−0.727***	−0.614***	−0.563***	−0.337**	−0.721***
LDL (mmol/L)	0.652***	0.597***	0.433***	0.228**	0.427***	0.767***
FPG (mmol/L)	0.340**	0.261**	0.432***	0.296***	0.675***	0.471***
2hPG (mmol/L)	0.550***	0.277***	0.198	0.287***	0.300*	0.162
FINS (μIU/mL)	0.281**	0.195*	0.370***	0.122	0.581***	0.463***
2hINS (μIU/mL)	0.279**	0.286***	0.696***	0.182*	0.109	0.172*
HbA1c (%)	0.253*	0.254**	0.274*	0.213*	0.265*	0.278**
HOMA-IR	0.326**	0.225**	0.427***	0.175*	0.677***	0.584***
HOMA-β	−0.007	−0.016	0.014	−0.084	0.208	−0.007

Data were analysed after adjusting for age, SBP and DBP. Variables with skewed distributions were log-transformed before statistical analysis.

*p<0.05, **p<0.01, ***p<0.001.

2hINS, 2 hour postload serum insulin; 2hPG, 2 hour postload plasma glucose; DBP, diastolic blood pressure; FPG, fasting plasma glucose; FINS, fasting serum insulin; HbA1c, glycated haemoglobin A1c; HDL, high-density lipoprotein; HOMA-IR, homeostatic model assessment of insulin resistance; LDL, low-density lipoprotein; SBP, systolic blood pressure; TC, total cholesterol; TG, triglyceride

### Risk of pre-diabetes and diabetes according to ApoB/ApoA-I ratio

The ApoB/ApoA-I ratios were further divided into tertiles and the first tertile was regarded as the reference group. In men, the risk of pre-diabetes in tertile 2 was 1.60-fold higher than in the first tertile (RR 1.602, 95% CI 1.080 to 2.122, p<0.05). However, this association disappeared after adjusting for age, SBP, DBP and TC. In parallel with pre-diabetes, the association of ApoB/ApoA-I ratio with diabetes also disappeared after adjusting for the aforementioned confounding factors in men (table 3).

**Table 3:** The risk of pre-diabetes and type 2 diabetes according to tertiles of apolipoprotein B/apolipoprotein A-I (ApoB/ApoA-I) ratio in men

		T1 (0–0.5703)	T2 (0.5704–0.7723)	T3 (≥0.7724)
Pre-diabetes	n, cases/participants	32/102	34/90	24/72
	Model 1	1	1.602 (1.080 to 2.122)*	1.427 (0.897 to 1.997)
	Model 2	1	1.381 (0.868 to 1.943)	1.160 (0.670 to 1.758)
	Model 3	1	1.327 (0.813 to 1.905)	1.113 (0.599 to 1.769)
				
Type 2 diabetes	n, cases/participants	18/102	31/90	26/72
	Model 1	1	2.461 (1.506 to 3.510)**	2.394 (1.422 to 3.485)**
	Model 2	1	2.035 (1.152 to 3.126)*	1.901 (1.034 to 3.022)*
	Model 3	1	1.643 (0.865 to 2.723)	1.074 (0.481 to 2.100)

Data are presented as RR (95% CI).

Model 1: unadjusted.

Model 2: adjusted for age, SBP, DBP.

Model 3: adjusted for Model 2+ TC.

*p<0.05, **p<0.01, ***p<0.001.

DBP, diastolic blood pressure; RR, relative ratio; SBP, systolic blood pressure; TC, total cholesterol.

Nevertheless, the associations between the ApoB/ApoA-I ratio and pre-diabetes or diabetes risk in women were more obvious than in men (table 4). In women, the risk of pre-diabetes was increased across the tertile of the ApoB/ApoA-I ratio (T2: RR 1.568, 95% CI 1.119 to 2.044, p<0.01; T3: RR 2.221, 95% CI 1.728 to 2.647, p<0.001). After adjusting for age, SBP, DBP, BMI and other lipid levels, the association between the ApoB/ApoA-I ratio and pre-diabetes was attenuated but still significant in T3 group (RR 2.186, 95% CI 1.376 to 2.842, p<0.01). Moreover, the crude RRs and 95% CIs of diabetes in tertile 2 to 3 were 3.943 (95% CI 2.540 to 5.500, p<0.001) and 5.940 (95% CI 4.353 to 7.272, p<0.001), respectively. After further adjusting for confounding factors, the risk of diabetes in the top tertile of the ApoB/ApoA-I ratio was still 3.65-fold higher than in the bottom tertile (RR 3.651, 95% CI 1.685 to 6.099, p<0.01).

**Table 4:** Risk of pre-diabetes and type 2 diabetes according to tertiles of apolipoprotein B/apolipoprotein A-I (ApoB/ApoA-I) ratio in women

		T1 (0–0.5703)	T2 (0.5704–0.7723)	T3 (≥0.7724)
Pre-diabetes	n, cases/participants	40/141	48/153	62/171
	Model 1	1	1.568 (1.119 to 2.044)**	2.221 (1.728 to 2.647)***
	Model 2	1	1.317 (0.876 to 1.826)	2.009 (1.472 to 2.503)***
	Model 3	1	1.478 (0.898 to 2.131)	2.186 (1.376 to 2.842)**
Type 2 diabetes	n, cases/participants	15/141	54/153	78/171
	Model 1	1	3.943 (2.540 to 5.500)***	5.940 (4.353 to 7.272)***
	Model 2	1	3.405 (2.078 to 5.001)***	5.439 (3.767 to 6.939)***
	Model 3	1	3.115 (1.598 to 5.126)**	3.651 (1.685 to 6.099)**

Data are presented as RR (95% CI).

Model 1: unadjusted.

Model 2: adjusted for age, SBP, DBP, BMI.

Model 3: adjusted for model 2+TC, TG, HDL-C, LDL-C.

*p<0.05, ** p<0.01, *** p<0.001.

BMI, body mass index; DBP, diastolic blood pressure; HDL-C, high-density lipoprotein cholesterol; LDL-C, low-density lipoprotein cholesterol; NGT, normal glucose tolerance; RR, relative ratio; SBP, systolic blood pressure; TC, total cholesterol; TG, triglyceride.

### Prevalence of pre-diabetes and diabetes according to tertile of apoB/apoA-I ratio

**Figure 1:**
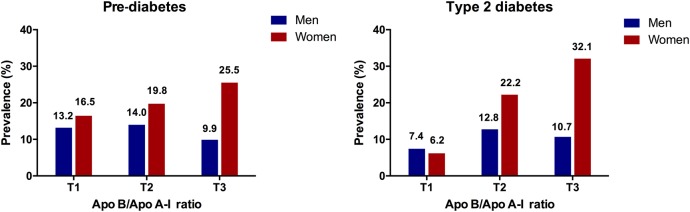
Prevalence of pre-diabetes and diabetes according to tertiles of apolipoprotein B/apolipoprotein A-I (ApoB/ApoA-I) ratio. The numbers above the bars mean the prevalence of each outcome, respectively. T1, tertile 1; T2, tertile 2; T3, tertile 3.

As shown in figure 1, the prevalence of pre-diabetes and T2DM in women were increased in sequence from the bottom to the top tertiles of the ApoB/ApoA-I ratio (for pre-diabetes: T1:16.5%, T2:19.8%, T3:25.5%, p=0.014; for diabetes, T1:6.2%, T2:22.2%, T3:32.1%, p<0.001). However, the prevalence of pre-diabetes and diabetes in men were higher in T2 than in T1 and T3 (for pre-diabetes: T1:13.2%, T2:14.0%, T3:9.9%, p>0.05; for diabetes, T1:7.4%, T2:12.8%, T3:10.7%, p>0.05).

## Discussion

In the current cross-sectional study, we observed strong positive associations between the ApoB/ApoA-I ratio and the risk of pre-diabetes and diabetes in women, independent of traditional metabolic risk factors. However, the associations in men were insignificant after adjusting for potential confounding factors. The ApoB/ApoA-I ratio was closely correlated with other lipid parameters and insulin resistance both in men and women.

It is well known that the ApoB/ApoA-I ratio is a better predictor of cardiovascular risk than other conventional lipid indices.^12^ However, only a few studies have shown the associations between apolipoprotein levels and the risk of diabetes. Hwang *et al*^13^ indicated that the ApoB/ApoA-I ratio is an effective predictor of T2DM in the Korean population. The ApoB/LDL-C ratio has been associated with T2DM in a population-based study of Turkish adults^14^ and ApoB in the Aboriginal Canadian population.^15^ Our results consistently suggested that the ApoB/ApoA-I ratio was associated with diabetes and pre-diabetes in Chinese women. Furthermore, the ApoB/ApoA-I ratio was closely correlated with TG, TC, HDL-C, LDL-C, FPG, HbA1C and HOMA-IR, which is in accordance with previous studies.^10^ ^16^

Apolipoproteins regulate the synthesis and metabolism of lipoprotein particles and stabilise their structures. Hence, it is not unexpected that the ApoB/ApoA-I ratio was closely related with TG, TC, HDL-C and LDL-C. Apolipoprotein B and A-I are the major protein moieties of LDL and HDL, respectively. The level of ApoB reflects the number of potentially atherogenic particles.^17^ Meanwhile, ApoA-I plays an important role in removing excess cholesterol from tissues and incorporating it into HDL for reverse transport to the liver, forming the basis for atheroprotective events.^18^ ^19^ Thus, the ApoB/ApoA-I ratio reflects the balance of atherogenic and atheroprotective particles, so the higher the level, the higher the tendency of cholesterol deposition, and consequently the higher the risk of CVD.^20^

Development of hyperglycaemia is closely associated with lipid disturbances.^21^ ^22^ In diabetic dyslipidaemia, ApoB-containing lipoprotein particles undergo compositional changes, including the increased formation of sd-LDL and large VLDL (VLDL-1) particles.^23–25^ These features were present in up to 50% of T2DM patients^26^ ^27^ and even in pre-diabetic patients with insulin resistance but NGT.^28^ This relationship has been demonstrated by several prospective studies, which indicated that increased levels of TG, sd-LDL, VLDL-1 and small HDL particles are closely associated with incident diabetes, whereas elevated levels of large HDL particles and Apo A-I are negatively associated with diabetes.^29–31^ In our study, we found the levels of ApoB were significantly increased in subjects with diabetes and pre-diabetes while the levels of ApoA-I showed the opposite trend across the spectrum of NGT, pre-diabetes and diabetes. As a consequence, the ratio of ApoB/ApoA-I was positively associated with pre-diabetes and diabetes. Moreover, this ratio was significantly correlated with insulin resistance. Previous studies have demonstrated that the ApoB/ApoA-I ratio is an independent predictor of insulin resistance in US non-diabetic subjects^32^ and Chinese obese subjects.^33^ A possible explanation for the positive association between the ApoB/ApoA-I ratio and insulin resistance could be that both ApoB and insulin resistance are linked to an inflammatory state.^34^ However, detailed mechanisms interpreting this association need further exploration.

The mechanisms leading to the accumulation of triglyceride-rich lipoproteins (TRLs) in patients with T2DM have not been fully elucidated. Hogue *et al*^35^ demonstrated that the elevated ApoB48-containing TRLs of intestinal origin and ApoB100-containing TRLs of hepatic origin in diabetic subjects are due to the increased production rates and decreased catabolism of these particles. Furthermore, Duez *et al*^36^ showed that intestinal secretion of ApoB48-containing TRLs is increased in insulin-resistant subjects with hyperinsulinaemia.

The effects of gender on lipid and apolipoprotein metabolism have been reported in several studies. Anahnostis *et al*^37^ conducted a cross-sectional study in premenopausal and postmenopausal Caucasian women and men and they showed that ApoB concentrations were higher in men than in women. In women, the levels rose with age. ApoA-I concentrations are highest in postmenopausal women and lowest in men. Li *et al*^38^ observed that postmenopausal status resulted in higher levels of all ApoB-containing lipoproteins. Hence, the risk of CVD was increased in women after menopause. The mechanisms of the gender effects on lipoprotein and apolipoprotein metabolism are not clear, but may be related to sex hormone changes. Some authors have reported that serum oestrone or oestradiol levels are positively correlated with HDL-C and TG and inversely associated with TC and LDL-C.^39^ ^40^ Androgen excess in premenopausal women, as is the case in polycystic ovary syndrome, has been associated with increased TG and sd-LDL particles, as well as reduced HDL-C.^41^ In addition, hyperandrogenemic women demonstrate increased insulin resistance and incidence of CVD.^42^ However, some other studies have reported conflicting results about the effects of oestrone and androgen.^43^ ^44^

In our study, it is noticeable that the gender difference was not significant in the NGT groups. In the pre-diabetic and diabetic groups, the levels of ApoB were increased in women, as were the ApoB/ApoA-I ratios. The results might be interpreted as indicating that age, gender, hormone levels, glucose and lipid levels exist in a complicated relative network and the corroborative effects of hormone and glucose status may accentuate the gender difference. We found the association between ApoB/ApoA-I ratio and the risk of diabetes was still significant in women after adjusting for conventional factors. Unfortunately, women were not further divided into premenopausal and postmenopausal groups due to the lack of information on menopausal status. However, from the median age of women in the pre-diabetic and diabetic groups (56.0 years old), we may make an inference that a great number of these women were in the postmenopausal status, which may, to some extent, explain the higher levels of ApoB in the women groups and also the higher risk of pre-diabetes and diabetes in women. In men, hormonal changes in andropause are not as recognisable as in female menopause^45^ ^46^ but they could also have an influence on our data. The risk of diabetes in men could also be derived from various factors, such as work stress, smoking and drinking, and the lack of physical exercise. The number of male participants included in this study was relatively small. These factors may be responsible for the lack of significant association between the ApoB/ApoA-I ratio and diabetes risk in men.

There were some limitations in our study. First, it was performed using a cross-sectional design and did not control for potential biases from diet, physical activity, smoking and drinking history. Second, women were not further divided according to menopausal status. Therefore, a prospective and well-controlled study would be needed to elucidate the associations of ApoB/ApoA-I ratio with diabetes and pre-diabetes risk.

In conclusion, our findings indicate positive associations between the ApoB/ApoA-I ratio and the risk of pre-diabetes and diabetes in Chinese women, independent of traditional metabolic risk factors. However, the associations in men were insignificant after adjusting for potential confounding factors. Collectively, the results of this study provide valuable evidence for a better understanding of the ApoB/ApoA-I ratio in detecting pre-diabetes and diabetes risk in the Chinese population, especially in women. Therefore, we propose the possibility of applying this ratio for risk assessment in Chinese women in epidemiologic investigations or routine clinical practice.
